# Similar evolutionary potentials in an obligate ant parasite and its two host species

**DOI:** 10.1111/j.1420-9101.2010.02223.x

**Published:** 2011-04

**Authors:** P S Pennings, A Achenbach, S Foitzik

**Affiliations:** Department Biology II, Ludwig-Maximilians-Universität MünchenPlanegg-Martinsried, Germany

**Keywords:** G_ST_, host–parasite coevolution, Jost D, maximum likelihood methods, population genetics, social insects

## Abstract

The spatial structure of host–parasite coevolution is shaped by population structure and genetic diversity of the interacting species. We analysed these population genetic parameters in three related ant species: the parasitic slavemaking ant *Protomognathus americanus* and its two host species *Temnothorax longispinosus* and *T. curvispinosus.* We sampled throughout their range, genotyped ants on six to eight microsatellite loci and an MtDNA sequence and found high levels of genetic variation and strong population structure in all three species. Interestingly, the most abundant species and primary host, *T. longispinosus,* is characterized by less structure, but lower local genetic diversity. Generally, differences between the species were small, and we conclude that they have similar evolutionary potentials. The coevolutionary interaction between this social parasite and its hosts may therefore be less influenced by divergent evolutionary potentials, but rather by varying selection pressures. We employed different methods to quantify and compare genetic diversity and structure between species and genetic markers. We found that Jost D is well suited for these comparisons, as long as mutation rates between markers and species are similar. If this is not the case, for example, when using MtDNA and microsatellites to study sex-specific dispersal, model-based inference should be used instead of descriptive statistics (such as *D* or G_ST_). Using coalescent-based methods, we indeed found that males disperse much more than females, but this sex bias in dispersal differed between species. The findings of the different approaches with regard to genetic diversity and structure were in good accordance with each other.

## Introduction

Host–parasite relationships are among the most widespread of all species interactions and important for the ecology and evolution of the interacting species. Host–parasite interactions can lead to coevolutionary arms races, where patterns of local adaptation can change over time and space. This spatial structure is often referred to as a geographic mosaic of coevolution (Thompson, 1994). We are interested in the coevolution of an obligate social parasite and its two host species and specifically in its spatial dimension. We use population genetic markers (microsatellites and MtDNA sequences) and explore different analytical methods to gain a better understanding of the coevolutionary dynamics of this host–parasite system. For many parasites, dispersal is tightly linked to host population structure either because parasites are transmitted directly from host to host or because the transmission stages can not survive long outside the host. This leads to the structure of host populations shaping parasite population structure, which has been indeed found in a number of parasite host systems (Nejsum *et al.*, 2005; Prugnolle *et al.*, 2005; Ren *et al.*, 2008).

Theory predicts that relative levels of gene flow and population sizes of hosts and parasites determine their evolutionary potential and are therefore among the main determinants of the coevolutionary dynamics (Lively, 1999; Gandon & Michalakis, 2002). Given that migration rates are still moderate enough to permit population differentiation, the opponent with higher influx of new alleles through migration or mutation is expected to be locally adapted. Comparative studies of host and parasite population structure and genetic diversity have been conducted in various study systems, with very diverse outcomes. In different snail-trematode systems, population structure was consistently more pronounced in the snail host than in the parasite (Dybdahl & Lively, 1996; Davies *et al.*, 1999; Keeney *et al.*, 2009). The amount of genetic diversity was either higher for the host (Dybdahl & Lively, 1996), or for the parasite (Keeney *et al.*, 2009) or it did not differ (Davies *et al.*, 1999). Parasites in these systems appear to benefit from higher gene flow and albeit they were not always more genetically diverse, they were repeatedly reported to be locally adapted (Dybdahl & Lively, 1995; Manning *et al.*, 1995; Mukaratirwa *et al.*, 1996). A study on a parasitic plant also found stronger population structure for the host plant than for the parasite, similar levels of genetic diversity and local adaptation for the parasite (Mutikainen & Koskela, 2002). Unfortunately, it is not possible to compare structure and diversity between hosts and parasites directly, if different genetic markers are used for the different species, because the reported F_ST_ measures depend on levels of diversity (Jost, 2008).

Social parasites of social insects are usually closely related to their hosts and are likely to have similar mutation and migration rates and comparable population sizes. Indeed, two recent studies on comparative population genetics in social parasites and their hosts, found no significant differences in either diversity or structure between hosts and parasites (Hoffman *et al.*, 2008; Foitzik *et al.*, 2009b). On the other hand, two other studies detected stronger population structure for the parasites and higher genetic diversity in the hosts (Trontti *et al.*, 2006, Vepsalainen *et al.*, 2009). Social parasites and their hosts are interesting study systems for host–parasite interactions, because due to their relatedness, the main difference between host and parasite species is their life style, as host and parasite respectively. An additional advantage is that the same molecular markers can be used for the host and the parasite alike.

We study a host-social parasite system from the north-eastern United States and Canada where the two host species and their shared social parasite are closely related ant species (Emery, 1909; Beibl *et al.*, 2005). The host species are *Temnothorax longispinosus* and *T. curvispinosus*, small ants which nest in acorns, hickory nuts or twigs on the forest floor. The parasite is the slavemaking ant *Protomognathus americanus*, which depends on enslaved host workers for all routine colony tasks such as brood care and foraging. This obligate social parasite exerts strong selection pressures on its hosts through frequent and destructive slave raids (Foitzik & Herbers, 2001). The host species display anti-parasite defence strategies such as enemy recognition, fighting and evacuation strategies and slave rebellion (Alloway, 1980; Foitzik *et al.*, 2001, Brandt and Foitzik, 2005; Achenbach & Foitzik, 2009) and these defences may have triggered parasite counter-adaptations (Brandt *et al.*, 2005).

In an earlier population genetic study, mainly based on MtDNA sequences, we found that the parasite *P. americanus* has higher levels of gene flow between sites, but overall less genetic diversity than its main host species *T. longispinosus* (Brandt *et al.*, 2007). In this study, we conducted a more comprehensive population genetic study to take a fresh look at genetic diversity and the population structure of the parasite and its two host species. We used more genetic markers (eight microsatellite loci vs. four in the previous study), studied more individuals (781 independent individuals from separate colonies vs. 184 in the previous study) and conducted additional and more sophisticated genetic analyses. We expect to find the highest genetic diversity in *T. longispinosus*, because it has the highest nest densities, followed by the second host species *T. curvispinosus* and then the social parasite *P. americanus*, which has only a tenth of the density compared with its hosts. Additionally, based on earlier data (Brandt *et al.*, 2007) and the biology of the species, we expect that the facultatively polygynous host species, where queens can return to the mother nest after mating, are more structured than the monogynous parasite species, *P. americanus*, where young queens found new colonies after dispersal and mating flights.

Our research objectives are, firstly, to compare genetic diversity and population structure between the three species and interpret these differences in the light of coevolution. Secondly, we aim to compare different methods which are available to study diversity and structure. For comparing genetic diversity between populations, species and loci, we focus on the effective number of alleles, which is a better measure of diversity than heterozygosity if the population-scaled mutation rate is high. To study population structure, we calculate D statistics (Jost, 2008), which are based on the effective number of alleles and are therefore better suited for highly variable markers. We apply a permutation test to ensure that *D*-values are significantly different from expectations under panmixia. To disentangle the effects of mutation and migration, we use two coalescent-based approaches to estimate population-wide mutation and migration rates.

Because the *D* statistic has not previously been applied to sequence data, we explore the effect of sequence length on *D*. For the MtDNA, we also use a test that takes into account genetic distances, because this may unveil different features of population structure. In addition, we analyse our data with the program Structure, which uses multi-locus genotypes to assign individuals to clusters (Pritchard *et al.*, 2000; Falush *et al.*, 2003).

## Methods

### Sampling sites and data collection

**Fig. 1:**
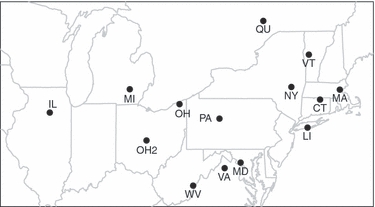
Collection sites in the United States of America and Canada. The geographic position of the collection sites can be found in Table 1.

Colonies of the parasite *P. americanus* and its hosts *T. longispinosus* and *T. curvispinosus* were collected between 2002 and 2008 in 13 locations throughout the northwestern United States and at one site in Canada (Table 1, Fig. 1). Ants were found in acorns, nuts and twigs on the floor of deciduous forests.

**Table 1:** Number of colonies sampled per species at each of the study sites. For geographic position of sampling locations see also Fig. 1

		Location		*T. longispinosus*		*T. curvispinosus*		*P. americanus*	
					
Abbreviation	Town or park	North	West	Microsatellites	Mt DNA	Microsatellites	Mt DNA	Microsatellites	Mt DNA
CT	Schenipsit, Connecticut	41.95	72.40		4*		5		10
IL	Ottawa, Illinois	41.37	88.83	50	6	48			
LI	Heckscher SP, Long Island	40.70	73.16				2*		2*
MA	Belmont Rock Meadows Park, Boston, Massachusetts	42.40	71.20		5		3*	4*	9
MD	Maryland	39.13	77.23	2*	1*	42		3*	2*
MI	Hell, Michigan	42.42	83.97	3*	2*	48			
NY	Huyck Pres., Rensselaerville New York	42.52	74.15	50	7			50	14
OH	Harpersfield, Ohio	41.75	80.95	50	4*	50	5	50	20
OH2	Kraus Wild. Preserve, Ohio	40.12	82.95			46		12	
PA	Elliot State Park, Pennsyl.	41.10	78.52	33	3*	10	3*		
QU	Gatineau, Quebec (Canada)	45.48	75.85		2*				
VT	East Middlebury, Vermont	38.88	78.20	32	9			2*	4*
VA	Shenandoah NP, Virginia	43.97	73.08	2*		44	3*	1*	1*
WV	Watoga SP, West Virginia	38.10	80.12	50	7	50	15	49	16

* Not used for most analysis, see Results section.

For microsatellite analysis, DNA was extracted from a total of 272 *T. longispinosus*, 338 *T. curvispinosus* and 171 *P. americanus* workers. Each worker came from a different nest (for sample size per location see Table 1). DNA was extracted using Puregene DNA extraction kit, Gentra System. Eight highly variable microsatellite loci were amplified with PCR using the primers L4 (Giraud *et al.*, 1999), L5 and L18 (Foitzik *et al.,* 1997), LXA GT1 (Bourke *et al.*, 1997), LX GT218 and LX GT223 (Hamaguchi *et al.*, 1993), MS86 (Azuma *et al.*, 2005) and Myrt 3 (Evans, 1993). For *P. americanus* the loci GT223 and MS86 did not amplify reliably and were not used. The polymerase chain reactions were performed as described in Foitzik *et al.* (2009b).

For the MtDNA analysis, we amplified and sequenced two overlapping DNA fragments covering about 1400 bp of the mitochondrial Cytochrome Oxidase I and II genes, using the methods described in Brandt *et al.* (2007). We added new sequences to enlarge the dataset from Brandt *et al.* (2007) to a total of 50 (22) *T. longispinosus*, 36 (22) *T. curvispinosus* and 78 (60) *P. americanus* individuals (in brackets are the number of individuals that were already used in Brandt *et al.*, 2007) (for sample sizes per location see Table 1).

### Data analysis

For the microsatellite data, the observed allele lengths were rounded to integers (binned) using the software TANDEM (Matschiner & Salzburger, 2009). Summary statistics were calculated using R (R Development Core Team 2005), with the packages *seqinr* (Charif & Lobry, 2007), APE (Paradis *et al.*, 2004) and *fields* (Furrer *et al.*, 2009). The microsatellite data were tested for heterozygote deficiency (test for H–W equilibrium, using a *U* test (Rousset & Raymond, 1995) and for linkage disequilibrium between loci (Fisher exact test for each pair of loci across all locations) using genepop (webversion: http://genepop.curtin.edu.au).

### Genetic diversity

To compare levels of genetic variation between species, locations and loci, the effective number of alleles, *n*_e_, was used, which is the reciprocal of homozygosity (*J*): 
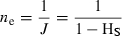
(1). H_S_ was calculated following Nei & Chesser (1983). We prefer *n*_e_, because, unlike heterozygosity (H), it changes linearly with diversity. If a population goes from 4 to 2 to 1 alleles (always at equal frequencies), heterozygosity goes from 0.75 to 0.5 to 0 (first a decrease of 33% and then a decrease of 100%, although in both cases half of the alleles are lost). The effective number of alleles would go from 4 to 2 to 1, which better reflects genetic diversity (see Jost, 2008).

To test whether species, location or locus influenced *n*_e_, we fitted a linear model with *n*_e_ as response variable and location, species and locus as explanatory variables using R. Separate analyses were performed for the microsatellite loci and MtDNA. We did not allow for interactions. For the linear model, we had to make the assumption that samples from different locations are independent, which is not strictly the case as they are related by genealogy. We repeated the same analysis with explanatory variables latitude and longitude.

### Population structure

We use several methods to test for population structure in the three species. First of all, we use the *D* and G_ST_ statistics to quantify differentiation for both microsatellite and MtDNA sequence data and we apply a permutation test to test for significance. We then discuss the influence of sample sizes and the length of the DNA sequence. We quantify differentiation using genetic distances between MtDNA sequences and determine whether there is a pattern of isolation-by-distance. Finally, we apply the program Structure (Pritchard *et al.*, 2000; Falush *et al.*, 2003) to the microsatellite data to detect higher level geographic structure and compare the outcomes with the *D* analysis.

#### D and G_*ST*_ analysis

Standard F statistics and their relatives, such as G_ST_, greatly underestimate differentiation when heterozygosity is high, which is usually the case for microsatellite markers (Hedrick, 2005; Jost, 2008). When heterozygosity is high, G_ST_ is automatically low, even when populations carry completely distinct sets of alleles. We therefore also calculated the *D* statistic, which was proposed by Jost (2008), and which is independent of within population diversity.

Jost *D* is usually written as follows:
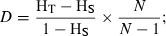
(2) (where *N* is the number of sampled subpopulations, H_T_ is heterozygosity over all samples, H_S_ is mean heterozygosity within the subpopulation samples). To get an intuitive understanding of *D*, it is useful to rewrite eqn 2 in terms of effective number of alleles (*n*_e_).
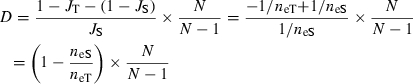
(3)

From eqn 3, one can see that *D* depends on the ratio between *n*_eS_ and *n*_eT_. In the finite-island model, *D* depends on the mutation rate and the migration rate between any two subpopulations (see eqn 22 in Jost, 2008). If mutation rates are expected to be the same between two species or loci, differences in *D* can be interpreted as differences in migration rates.

#### Permutation test for significance of *D* and G_*ST*_

We tested the significance of the *D* and G_ST_ values with a simple permutation test (as described, for example, in Hudson *et al.*, 1992), using 1000 randomized datasets. For each randomized dataset, individuals are redistributed over the subpopulations in such a way that all individuals are used (no replacement) and the sizes of the population samples do not change. We used individuals, and not alleles, as the unit for permutation, because the population samples were not in Hardy–Weinberg equilibrium, so using individuals is the conservative choice. From each randomized dataset, *D* and G_ST_ are calculated. The *P*-value of the test is the percentage of *D* (or G_ST_) values from the randomized datasets that are higher than or equal to the real *D* (or G_ST_) value. We used the same randomized datasets for *D* and G_ST_ and the resulting *P*-values are exactly the same for *D* and G_ST_. Gerlach *et al.* (2010) also noted that statistical significance did not differ between *D* and G_ST_. Note that this permutation test is not the same as the bootstrapping procedure as implemented in the web-based smogd software (Crawford, 2009). The bootstrapping procedure creates new datasets consisting of subsets of the original data, but without redistributing the individuals over the locations.

#### Statistical issues: sample sizes and length of MtDNA sequence

Simulations show that the power to detect population differentiation (with *D* or G_ST_) is reduced when some of the samples are very small (Hudson *et al.*, 1992). We therefore removed all samples with fewer than five individuals from the analysis (see Table 1).

For the MtDNA sequences, we calculated *D* and G_ST_ based on alleles. Longer sequences usually create more different alleles, and the values of *D* and G_ST_ will be influenced by the length of the DNA sequence which is used for a study. We studied this effect by analysing parts of the DNA sequence, as if we had only sequenced a shorter fragment. For each fragment length (100, 300, 500, 700, 900, 1100 bp), we took a fragment from our dataset, starting at a random location in the sequence and calculated *D*, G_ST_ and *P*-values. We repeated this 20 times for each fragment length and averaged over these 20 runs. For each randomly selected fragment, a *P*-value was estimated by 1000 permutations. We estimate the power to detect population structure as the percentage of these 20 fragments which showed a *P*-value of < 0.05.

#### Using genetic distances

The previous analyses of the microsatellites and MtDNA data are based on alleles, not genetic distance. When calculating *D*, there is no elegant way to include genetic distances, because it is based on the effective number of alleles.

In principle, one can calculate *D* for each nucleotide and then average over nucleotides, so that when more nucleotides have high *D* values, the average *D* value will be high. However, *D* per nucleotide is not very informative. For most nucleotides *D* will be 0, because any sequence which can be aligned will consist mostly of nucleotides which are monomorphic. Even for sites that are polymorphic, *D* will still be low because there are usually a maximum two states at each nucleotide. *D* is highest if each subpopulation carries unique alleles, but for single nucleotides this is not possible. In practice, this means that *D* per nucleotide must go down as more locations are sampled.

By contrast, genetic distances can be easily integrated in G_ST_ analyses by using heterozygosities (H_T_ and H_S_) per nucleotide and averaging over all nucleotides (H_Tnuc_ and H_Snuc_). These heterozygosities increase with genetic distance between individuals. Using H_Tnuc_ and H_Snuc_, one can calculate 
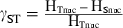
 (Nei, 1982). Note that γ_ST_ does not suffer from the same problems as G_ST_ does because there are usually only two and definitely not more than four allelic states per nucleotide, so heterozygosity cannot be high and is unlikely to reach a level where it does not increase (approximately) linearly with diversity. Significance can be estimated with the same permutation test as described before.

#### Isolation by distance

To test for isolation by distance, a Mantel test was performed over the whole dataset using the web based software ibdws (Jensen *et al.*, 2005) for both microsatellites and MtDNA. For the microsatellites, we used pairwise *D* values, for the MtDNA we used genetic distances (mean number of pairwise differences) directly.

#### Structure analysis

We analysed the microsatellite data with the program Structure 2.3.1 (Pritchard *et al.*, 2000; Falush *et al.*, 2003). We did the standard analysis with admixture, a burn in period of 50 000 iterations, then 100 000 iterations of the Markov chain. *K* (number of clusters) ranged from 1 to 6 for all species and we did four repeats per *K* value and used delta *K* (Evanno *et al.*, 2005) to decide on the ‘true’ value of *K*. To determine delta *K* we used *Structure Harvester* (http://taylor0.biology.ucla.edu/struct_harvest/). We compared the outcome of Structure with pairwise *D* values averaged over loci.

### Estimating population-wide mutation and migration rates

The previous analyses describe the observable pattern of population structure, which is created by processes such as mutation and migration. Ideally, we would like to infer the parameters of these underlying processes using a model based approach (Beaumont *et al.*, 2010). This is possible, in principle, using a likelihood or Bayesian framework, as implemented in programs such as migrate-n (Beerli & Felsenstein, 2001) or Migraine (Rousset & Leblois, 2007). Realistically, parameter estimation is only possible in a simplified model with fewer parameters than the natural system, for example by ignoring unsampled demes, which means that the estimates obtained may be biased by model misspecification. Some of these model misspecifications have been studied. Specifically Beerli (2004), Slatkin (2005) and Rousset & Leblois (2007) found that even if unsampled demes are ignored, it is often still possible to estimate well the product of *θ* (population-wide mutation rate) per subpopulation and *N*_T_ (the number of subpopulations).

**Fig. 6:**
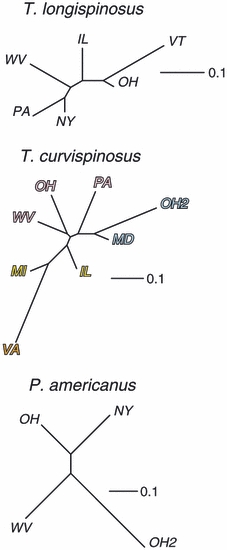
Neighbour-joining trees using pairwise *D* values. Pairwise *D* values were averaged over the eight microsatellite loci (six loci for *Protomognathus americanus*). Names at the tips of the trees correspond to the locations of the samples (see Table 1). The colours of the labels in Fig. 6b reflect the four clusters in b.

For the microsatellite data, we use *Migraine* (Rousset & Leblois, 2007), which is based on the importance sampling algorithm by De Iorio & Griffiths (2004a,b);, to estimate θ*N_T_ and the migration rate M for each locus and each species. We assume that our data are from a finite-island model (with equal population sizes in each subpopulation and equal migration rates between all subpopulations). Two aspects of our data make us confident that the finite-island model is not too far from reality for our species. (i) Our samples from different locations do not differ significantly in genetic diversity, which indicates that local population sizes are similar. (ii) Values of pairwise differentiation are similar between all locations (see Fig. 6), which suggests that migration rates are relatively symmetric. We ignore unsampled subpopulations and assume that mutations follow a K-allele model and that population size is constant. We run Migraine for each locus separately with the following settings: the shape parameter, *g*, is fixed at 1, so that migration is independent of distance and the model is a finite-island model. We first estimated likelihoods by 10 runs at each of 2000 points in the parameter range 2Nμ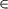 [0,40] and 2N*m*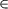 [0,100], and in a second round, 30 runs at 512 points in a range of high likelihoods as determined by the program in the first round. In one case (locus Myrt3 for *T. longispinosus*), the original range did not include the whole confidence interval, so we reran with 2N*m*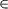 [0,100]. The output of Migraine for the mutation rate is divided by the number of samples, *N*, so we have to multiply the output by *N* to get θ*N_T_. For the migration rate, the output is also divided by *N*, which we use as is.

For the MtDNA data, we run migrate-n (Beerli & Felsenstein, 2001), which uses an MCMC algorithm to find maximum likelihood estimates. We assume a finite-island model. We ran the program with the following settings: we ran 10 short-chains, with 50 000 sampled trees, and one long chain with 1 000 000 sampled trees, a 100 step increment in both cases and a burn in of 100 000 steps. migrate-n also outputs an estimate of θ, whereas we are interested in θ*N_T_*.* We therefore multiply the estimated θ value with *N* (the number of subpopulations). Unlike Migraine, migrate-n estimates migration rates between all sampled populations (e.g. the migration rate from subpopulation 1 to subpopulation 2). We multiply the estimated migration rate with the number of neighbouring populations to get the total migration rate M.

To test whether θ*N_T_ or M are different between the species of interest, we fit a linear model with the estimated values for θ*N_T_ and M as response variables and species and locus as explanatory variables using *R*.

## Results

### Binning of the microsatellite data

The authors of *TANDEM* suggest that good loci have an average rounding error which is below 10% of the repeat size. Only three of the eight loci were below this threshold for *T. longispinosus* (Myrt3, GT223 and MS86), two of the six for *P. americanus* and also two of eight for *T. curvispinosus* were below this threshold (L5 and L18 for both species). Nevertheless, we decided to keep all loci in the analysis. For the demography and the population structure analysis, we found no differences between the high and the low quality loci. For the genetic diversity analysis, the data of only the high quality loci are not enough to make a comparison between the species, but also for this analysis, the results seem consistent over loci.

### Linkage disequilibrium and Hardy–Weinberg

Hardy–Weinberg equilibrium over all subpopulations and loci was rejected because of heterozygote deficiency for all three species (*P*< 0.0001). The underlying reason could be the presence of null-alleles and/or inbreeding. We proceeded to estimate the proportion of null-alleles for each species, locus and population, using the method by Chakraborty *et al.* (1992) under the assumption that heterozygote deficiency was entirely because of null-alleles. Averaging the proportion over populations gave us upper limits of the proportion of null-alleles for each locus and species which ranged from 0 to 0.39 with an average of 0.10. When performing the permutation test for *D* and G_ST_ we will therefore permute individuals and not alleles.

Significant linkage disequilibrium was found in all three species: *T. longispinosus* had six significant locus pairs (*P*< 0.01) out of 28 (L5 & GT1, L5 & L18, GT1 & L18, L5 & Myrt3, GT1 & Myrt3, L18 & Myrt3), *T. curvispinosus* seven of 28 (L5 & GT1, L5 & L18, L18 & Myrt3, L5 & GT223, Myrt3 & GT223) and *P. americanus* nine of 15 (L5 & GT1, L5 & L18, GT1 & L18, L5 & Myrt3, GT1 & Myrt3, L18 & Myrt3, L18 & GT218, Myrt3 & GT218, L18 & L4). This could mean that the different loci are not independent and not too much weight should be placed on evidence which comes from different loci.

### Genetic diversity

**Fig. 2:**
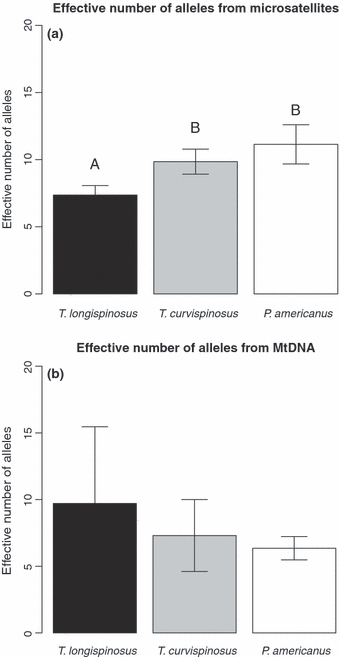
(a) Effective number of alleles (*n*_e_) for microsatellites. Values are averaged over populations and loci. Bars indicate standard errors. *Temnothorax longispinosus* has a lower effective number of alleles than the other two species. (b) Effective number of alleles (*n*_e_) for MtDNA sequences for all three species. Values are averaged over populations. Bars indicate standard errors.

The mean effective number of alleles (*n*_e_) averaged over loci and locations varied between 7 and 11 for the three species for the microsatellites (Table 2, Fig. 2a) and between 6 and 10 for the MtDNA data (Table 2, Fig. 2b). For the microsatellites, we found that *T. longispinosus* has significantly lower *n*_e_ than the other two species (*P*< 0.005). The different loci had different values of *n*_e_ (all *P*< 0.05), but the study sites were not significantly different (*P*= 0.98). We found no effect of longitude and latitude (*P*= 0.60). For the MtDNA dataset, we found no effect of species or location (all *P*> 0.05).

**Table 2:** Comparison of *n*_e_ (effective number of alleles) and H (heterozygosity) values for all loci (eight microsatellite loci and MtDNA) and species. For the MtDNA, we also report π (mean pairwise differences per nucleotide). In all cases, values are calculated within each population and averaged over all populations

Species	L5	GT1	L18	Myrt3	GT218	L4	GT223	MS86	Mean microsatellites	MtDNA
*T. longispinosus*										
*n*_e_	11.0	12.9	8.3	6.1	3.2	12.5	3.3	1.6	7.4	9.7
H	0.88	0.92	0.88	0.83	0.65	0.92	0.57	0.31	0.74	0.86
π										0.020
*T. curvispinosus*										
n_e_	4.8	22.2	7.6	11.9	4.8	15.4	9.4	2.9	9.8	7.3
H	0.75	0.95	0.86	0.91	0.74	0.90	0.86	0.50	0.81	0.76
π										0.005
*P. americanus*										
*n*_e_	10.7	16.4	11.3	13.2	4.3	10.9	–	–	11.1	6.4
H	0.91	0.91	0.90	0.92	0.66	0.89	–	–	0.86	0.83
π										0.010

### Population structure

#### G_ST_ and D

**Fig. 3:**
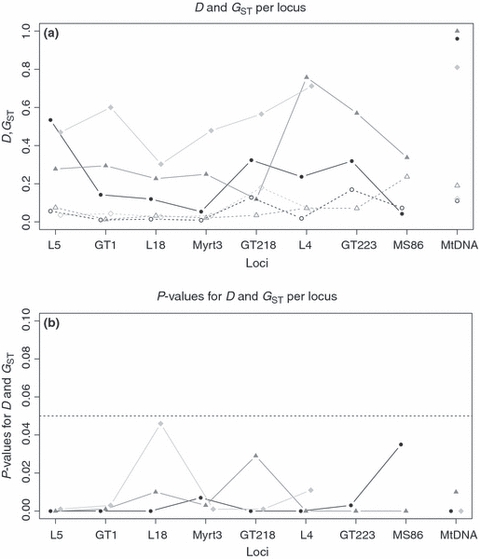
(a) *D* and *G*_ST_ values for the different microsatellite loci and all three species. Black circles: *T. longispinosus*, dark grey triangles: *T. curvispinosus*, light grey diamonds: *P. americanus*. Closed symbols: *D* values, open symbols *G*_ST_ values. (b) Significance of the *D* and *G*_ST_ values given in (a). Black circles: *T. longispinosus*, dark grey triangles: *T. curvispinosus*, light grey diamonds: *P. americanus*. Dashed line: 0.05 significance level. *P*-values are based on 1000 permutations and are the same for *D* and *G*_ST_ (see text for details).

G_ST_ values for the microsatellite loci were low for all three species, on average 0.03 for *T. longispinosus*, 0.07 for *T. curvispinosus* and 0.03 for *P. americanus,* as expected for highly variable loci. The values, however, were always higher than expected under panmixia (*P*-values between 0.001 and 0.034, Table 3 and Fig. 3a). Jost *D* values were much higher than the G_ST_ values: *T. longispinosus*: 0.25, *T. curvispinosus*: 0.35, *P. americanus*: 0.58. *D* values were significantly lower for *T. longispinosus* than for the other two species (*P*= 0.022), indicating a higher migration rate for this species. For the MtDNA data, both G_ST_ and *D* values were consistently higher than for the microsatellite loci (Table 3 and Fig. 3a). The *P*-values were below 0.05 for all species (Table 3 and Fig. 3b).

**Table 3:** Comparison of the *D* and *G*_ST_ values for all loci and species

Species	L5	GT1	L18	Myrt3	GT218	L4	GT223	MS86	Mean	mtDNA
*T. longispinosus*										
*D*	0.62	0.23	0.16	0.07	0.32	0.24	0.31	0.02	0.25	0.96
*G*_ST_	0.05	0.01	0.01	0.01	0.05	0.01	0.07	0.01	0.03	0.11
*P*-values	0.000	0.000	0.000	0.007	0.000	0.000	0.003	0.034		0.0001
*T. curvispinosus*										
*D*	0.28	0.29	0.23	0.25	0.12	0.76	0.57	0.34	0.35	1.00
*G*_ST_	0.07	0.01	0.03	0.02	0.03	0.07	0.07	0.24	0.07	0.19
*P*-values	0.000	0.001	0.010	0.003	0.029	0.000	0.000	0.001		0.010
*P. americanus*										
*D*	0.60	0.67	0.33	0.59	0.55	0.75	–	–	0.58	0.81
*G*_ST_	0.03	0.03	0.02	0.03	0.08	0.04	–	–	0.04	0.12
*P*-values	0.001	0.000	0.05	0.001	0.001	0.01	–	–		0.000

#### Effect of length of fragment for MtDNA

**Fig. 4:**
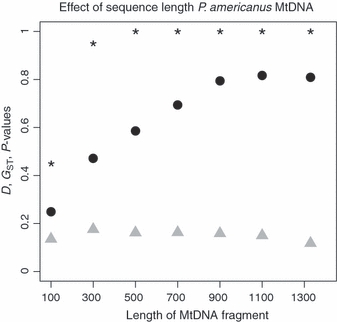
Effect of the length of the MtDNA sequence on *D*, *G*_ST_ and the power to detect population structure using the data from *Protomognathus americanus*. Circles: *D* values, triangles: *G*_ST_ values, asterisks: power to detect population structure. Each datapoint for *D* and *G*_ST_ is based on 20 randomly chosen fragments of the total data. For each randomly selected fragment, significance was estimated by 1000 permutation. The power is the percentage of these 20 fragments which showed a *P*-value of < 0.05.

For short fragment lengths, *D* values were low and the permutation test often gave nonsignificant results. As an example, we show the result for *P. americanus* (Fig. 4). The results for the other species are similar (not shown). We show the average *D* and G_ST_ value for a given fragment length and the proportion of tests that gave a significant (*P*< 0.05) result. The number of base pairs that were needed for consistently significant results was 500 for *P. americanus,* 300 for *T. longispinosus* and 900 for *T. curvispinosus*.

From 300 to 1305 base pairs, G_ST_ slowly goes down because heterozygosity goes up when longer sequences are considered. *D* goes up with longer fragments, as it is more likely that mutations which make subpopulations differentiated are found. However, if the mutation rate increases even more (had we sampled an even longer fragment), the *P*-values would go up and power goes down (unpublished data). The reason is that if (almost) every individual carries a different allele, there is no power to detect population structure. This is not the case in our dataset.

#### Using genetic distances

We find that *T. longispinosus* shows the highest γ_ST_ (Table 4), which is significantly different from expectations under panmixia (*P*= 0.01), *T. curvispinosus* has a lower value of γ_ST_ which is borderline significant (*P*= 0.05). *P. americanus* has even lower γ_ST_, which is not significant (*P*= 0.25).

**Table 4:** Distance based measure of population differentiation

	γ_ST_	*P*-value
*T. longispinosus*	0.23	0.01
*T. curvispinosus*	0.18	0.05
*P. americanus*	0.03	0.25

#### Isolation-by-distance

We found no significant correlations between genetic and geographic distances in *T. longispinosus* or *P. americanus* for the microsatellite or the MtDNA data (*P*-values > 0.05). For *T. curvispinosus*, we found a negative correlation for the microsatellites: subpopulations that are further away from each other tend to have lower *D* values (*r* = −0.39, *P*= 0.04), but a positive one for the MtDNA (*r* = 0.99, *P*< 0.001). The latter, however, is based on only three subpopulations (OH, WV and CT).

#### Structure analysis

**Fig. 5:**
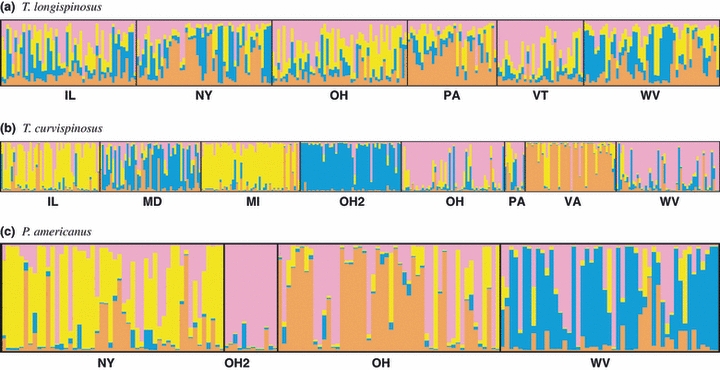
Structure analysis results for three species. Shown are the estimates of Q (estimated membership coefficient for each individual) for each cluster. The most probable number of genetic populations present in the data is *K* = 4 for *Temnothorax longispinosus* (a), *K* = 4 for *T. curvispinosus* (b) and *K* = 4 for *Protomognathus americanus* (c). The vertical lines are broken into coloured segments showing the proportion of each individual assigned to each of the inferred clusters. Names above the graph correspond to the state of the various sample sites (see Table 1).

For all three species, we find the highest delta *K* for *K* = 4 (Table 5, Fig. 5). For *T. longispinosus*, we did not detect meaningful structure and most individuals could not be convincingly assigned to either of the clusters (Fig. 5a). By contrast, *T. curvispinosus* is clearly structured, each of the subpopulations is relatively homogeneous, and some subpopulations cluster together (Fig. 5b). For *P. americanus*, we also find the highest delta *K* for *K* = 4, which is equal to the number of locations we have samples from. The clusters are not very clear, but subpopulations do seem to be differentiated (Fig. 5c).

**Table 5:** Results of the structure analysis. The Log likelihood of the data given *K* (the number of subpopulations), averaged over four independent runs is given. For the settings of the program, see text

*K*	*T. longispinosus*	*T. curvispinosus*	*P. americanus*
1	−8324	−12050	−4666
2	−8256	−11797	−4530
3	−8204	−11529	−4357
4	−8098	−11304	−4242
5	−8074	−11189	−4285
6	−7944	−11077	−4206

**Fig. 7:**
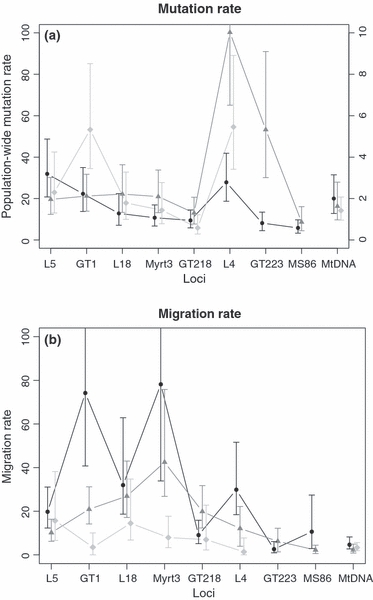
Estimated values of mutation and migration rates from the program Migraine and migrate-n for all three species. Black circles: *T. longispinosus*, dark grey triangles: *T. curvispinosus*, light grey diamonds: *P. americanus*. Arrows indicate 95% confidence intervals as estimated by the programs. (a) Population-wide mutation rates (θ*N_*T*_), (b) migration rates (*M*).

Using pairwise *D* values, we created neighbour-joining trees (Fig. 6), which can be compared with Structure outcomes. For the one species for which Structure detected higher level structure, *T. curvispinosus*, we find that that Structure and *D* agree quite well (see Figs 5b and 7b). Both Structure and D cluster OH2 and MD together, just like OH and WV. *D* clusters MI, IL and VA together, whereas Structure only clusters MI and IL together. However, when Structure is run for *K* = 3, it also consistently places VA with IL and MI (data not shown). The colours in the population labels in Fig. 6b are chosen to reflect the clustering in Structure (Fig. 5b).

### Estimating population size and migration rate

For the microsatellites, the maximum likelihood estimate for θ*N_T,_ averaged over loci as estimated with Migraine is 2.7 for *T. longispinosus*, 4.0 for *T. curvispinosus* and 6.3 for *P. americanus* (Fig. 7a)*.* Population-wide mutation rates (θ*N_T_) did not differ between species (*P*= 0.2), but locus had an almost significant effect (*P*= 0.05). The maximum likelihood estimates for M, averaged over loci, is 32.0 for *T. longispinosus*, 17.6 for *T. curvispinosus* and 6.5 for *P. americanus* (Fig. 7b). We found differences between species in migration rates (*P* = 0.02), although none of the one-by-one comparisons between the three species were significant. As expected, locus did not affect migration rate (*P*= 0.08).

For MtDNA, the maximum likelihood estimate for θ*N_T_ (per nucleotide), as estimated by migrate-n is 0.004 for *T. longispinosus*, 0.005 for *T. curvispinosus* and 0.003 for *P. americanus* (Fig. 7a). The maximum likelihood estimates for M are 1.1 for *T. longispinosus*, 1.1 for *T. curvispinosus* and 0.84 for *P. americanus* (Fig. 7b). The confidence intervals for the estimates of the three species strongly overlap, so that species differences were not apparent.

## Discussion

Our population genetic analyses reveal that all three interacting ant species have high genetic diversity and strongly structured populations. From these results and earlier evidence for strong reciprocal selection pressures (Foitzik *et al.*, 2001), we conclude that spatially structured coevolutionary arms races are expected, which could lead to local adaptation. Indeed, chemical analyses and field and laboratory manipulations demonstrated that the social parasite *P. americanus* is locally adapted to its host species (Achenbach & Foitzik, 2009; Foitzik *et al.*, 2009a). Behavioural experiments also revealed clear population differences, but no local adaptation in either host or parasite (Foitzik *et al.*, 2001; Brandt & Foitzik, 2004).

Overall, the three species resemble each other with respect to migration and mutation patterns and we conclude that they have similar evolutionary potentials. Adaptation not only depends on the availability of genetic variation, but also on the strength of selection. Therefore our results suggest that to gain a better understanding of who leads the coevolutionary arms race in this host–parasite system, we should focus on reciprocal selection pressures rather than on population genetics. In contrast to other comparative studies on host and parasite population genetics (Dybdahl & Lively, 1996; Keeney *et al.*, 2009; Trontti *et al.*, 2006; Vepsalainen *et al.*, 2009), the differences we find between our study species are very small. Similar levels of genetic diversity and structure between host and parasite was also found in two other social parasite systems of a slavemaking ant and a socially parasitic wasp (Hoffman *et al.*, 2008; Foitzik *et al.*, 2009b). In the social wasp system, neither host nor parasite was clearly structured, but sampling was performed on a much smaller scale than in our studies. In other host–parasite systems, large differences, often 10-fold, in either diversity or structure were found and this was also the case for two other host-social parasite systems (Trontti *et al.*, 2006; Vepsalainen *et al.*, 2009), in which the parasite was much less variable and more structured. Host-social parasite systems with similar levels of diversity and structure are therefore interesting exceptions, which are worth studying because the opponents are on equal footing in evolutionary terms (current study, Foitzik *et al.*, 2009b; Hoffman *et al.*, 2008).

Albeit the overall pattern was one of similarity, the species did differ from each other in some surprising aspects. For example, although *T. longispinosus* has the highest population densities, we find that it has lower genetic diversity at the microsatellite loci in the subpopulations. Moreover, we expected the parasite *P. americanus* to be less structured than the other two species, because it is strictly monogynous, but it turned out to be as structured as *T. curvispinosus* and more structured than *T. longispinosus* (at the microsatellite loci) and its estimated migration rates were lowest. Only the MtDNA analyses based on genetic distances revealed the expected pattern of structure in the host species but not in the parasite.

There are several limitations to the inferences we can make based on our data. Naturally, we have data from a limited number of subpopulations and a limited number of microsatellite loci. The mitochondrial genome is nonrecombining and therefore only ‘shows’ one genealogical history, which can be greatly influenced by chance events. In addition, we detected linkage disequilibrium and an excess of homozygotes. The presence of linkage disequilibrium means that different loci are not entirely independent, and evidence based on different loci should therefore be treated with caution. The excess of homozygotes could be attributed to null-alleles or inbreeding, which could make subpopulations look more differentiated than they really are. Keeping in mind these limitations, we will now focus on each of the species separately.

### T. longispinosus

At the microsatellite loci, we found a significantly lower effective number of alleles (*n*_e_) for the host species *T. longispinosus* compared with the other two species. The same trend (though not significant) was found for the estimates of θ*N_T_ by the software Migraine. This is surprising, given that *T. longispinosus* occurs at 10-fold higher densities than the parasite *P. americanus* (Herbers & Foitzik, 2002; Brandt & Foitzik, 2004) and is also more abundant in its native range than *T. curvispinosus* (Mackay, 2000). Possibly, for reasons unknown to us, only relatively few nests contribute to the next generation, leading to higher drift in *T. longispinosus* compared with the other species. The effective population size of Hymenopteran species is generally lower because of haplodiploidy and even more so if complementary sex determination leads to the production of diploid males (Zayed, 2004). Indeed, diploid males have been detected in *T. longispinosus*, but not in the two other study species (Foitzik *et al.*, 2004).

Another possible explanation for the lower genetic diversity in *T. longispinosus* is that the population has only recently grown to its current size. Such a population expansion leaves a specific genetic signature, which can be detected with the *k*-test of Reich & Goldstein (1998). We found indeed that the species *T. longispinosus* had eight negative *k* values (exact binomial test *P*= 0.008), which can be interpreted as evidence for a population size expansion in this species. In the other two species, we found no significant deviation from expectations (*T. curvispinosus*: six negative values out of eight, *P*= 0.29, *P. americanus*: four negative values out of six, *P*= 0.69). It is therefore likely that the *T. longispinosus* population has recently increased in size in which case the observed genetic diversity will reflect ancient population sizes, rather than current. The evolutionary potential, however, is determined largely by its current population size (Charlesworth, 2009, Karasov *et al.*, 2010). An empirical example for an increase in the evolutionary potential because of a recent population expansion stems from the comparative population genetic analyses of a host–parasite system between a fungal parasite and an introduced host plant (Linde *et al.*, 2010). Genetic diversity should therefore be used with caution to predict the evolutionary potential of a species.

*Temnothorax longispinosus* shows clear evidence for population structure, but at the microsatellite loci it has the lowest *D* values of the three species (Fig. 3a), and the highest migration rates (Fig. 7b). The Structure analysis confirms the low level of population differentiation in this species. The MtDNA sequence data for *T. longispinosus* show clear population structure and high diversity (Figs 2 and 3), similar to what was found in the previous study (Brandt *et al.*, 2007). The much clearer population structure for the MtDNA when compared with the microsatellite loci suggests that in this species most dispersal is done by males.

### T. curvispinosus

The host *T. curvispinosus* has lower population density than *T. longispinosus*, but higher diversity in the subpopulations. It also shows stronger population structure than *T. longispinosus* in every analysis: it has higher *D* values (Fig. 3a), lower maximum likelihood estimates of migration rates (Fig. 7b) and the Structure analysis shows clearer structuring (see Fig. 5). However, the subpopulations, which are clustered by Structure, are surprising: Northern Ohio and West-Virginia cluster together and Southern Ohio and Maryland. Geographically these clusters are not close to each other at all. When we test for isolation by distance, using pairwise *D* values (for the microsatellites) we find a negative correlation between geographic distance and *D* values; the populations that are further away tend to be closer genetically. This is clearly a surprising finding. However, other phylogeographic studies found that postglacial resettlement routes can lead to a divergent evolutionary history of close populations, for example, because they stem from different pleistocene refuges. For example, a population genetic analysis of the shrew *Sorex antinorii* detected two resettlement routes in the European Alps. These two genetic lineages came into secondary contact in the Rhone valley and neighbouring shrew populations are genetically clearly distinct (Yannic *et al.*, 2008). For our study species, more detailed population genetic analyses (including more populations) would be necessary to reconstruct similar phylogeographic patterns. In a previous study (Brandt *et al.*, 2007), we detected no significant population structure for *T. curvispinosus* at the microsatellite loci, but this is attributed to the fact that we only had samples from two locations which, in hindsight, turned out to have a very low pairwise *D* value (Northern Ohio and West Virginia).

### P. americanus

The social parasite *P. americanus* has a slightly smaller range than *T. longispinosus* and only 10% of its host density. Still, we find that it has as high diversity as *T. curvispinosus* and higher genetic diversity in the subpopulations than *T. longispinosus* for the microsatellites (see Fig. 2). It also has the highest maximum likelihood estimate of θ*N_T_ (Fig. 7a).

All three species conduct mating flights, but their reproductive biology differs. *P. americanus* colonies invariably contain a single queen, whereas both host species are facultatively polygynous. This means that in the two host species, some host queens can return to the mother nest and are re-adopted (and thus have zero dispersal). Strongly structured populations have been found for example in polygynous ant species of the genus *Myrmica* and *Formica* (Seppa & Pamilo, 1995; Sundstrom *et al.*, 2005). Moreover, an association between social organization and genetic structure has also been found for the ant *Petalomyrmex phylax*, which shows inter-populational differences in the degree of polygyny. (Dalecky *et al.*, 2007). In *P. americanus* queens always disperse and found new colonies by invading host nests. We therefore expected *P. americanus* to have higher migration rates, at least for the females, which would be reflected in the MtDNA. And indeed we found that this species has a lower *D* value for the MtDNA compared with the other two species. Besides, the genetic distance based test is not significant for *P. americanus*, whereas it is for the two facultatively polygynous host species, which fits with the expectation. However, the maximum likelihood estimates of the migration rate (from migrate-n, using the MtDNA data) were not different between the three species (see Fig. 7b).

Surprisingly, we found that *P. americanus* had the strongest population structure of the three species for the microsatellite loci (though *D* values were not significantly higher than those for *T. curvispinosus*) (Fig. 3a), and the lowest estimated migration rates (Fig. 7b). We hypothesize that this may be because dispersal could be more risky for *P. americanus,* because it needs a suitable abiotic habitat and a host colony to settle successfully in a new patch. The density of the species is also lower, so males may be less likely to find a mate if they disperse. If dispersal related death is higher, optimal dispersal rates are lower and effective dispersal rates may be even lower, leading to higher differentiation between subpopulations (Gros *et al.*, 2006). We recently found that in a European host-social parasite system, structure was also strongest for the parasite (Foitzik *et al.*, 2009b) and another recent study on a slavemaker species found even much stronger population structuring (Sanllorente *et al.*, 2010). This could mean that it is a general pattern that obligate social parasites exhibit low dispersal because this is a more risky endeavour for them.

### MtDNA vs. microsatellites

We find consistently higher G_ST_ and *D* values for MtDNA than microsatellites, as did many other population genetic studies on ants (Doums *et al.*, 2002; Clemencet *et al.*, 2005; Brandt *et al.*, 2007; Goropashnaya *et al.*, 2007). A common conclusion from this finding was that ant queens disperse less than males. However, we think that this conclusion based only on different levels of differentiation is incorrect, because such a difference could also be caused by differences in mutation rates between the marker systems. Indeed, our results show that the mutation rates are very different for the two marker systems. If we multiply the estimated per nucleotide population-wide mutation rates for the MtDNA (from migrate-n) with the number of nucleotides we have sequenced, we find an average population wide mutation rate of 22 for the MtDNA locus, whereas the microsatellite loci have an average population wide mutation rate of 4. The per-individuum mutation rates are even more different than this comparison suggests, because population size for MtDNA is 4-fold lower than for nuclear markers. This means that the differences in *D* values between the marker systems could be entirely attributed to differences in mutation rates.

If one is interested in sex-specific dispersal, one could compare the maximum likelihood estimates of migration rates between marker systems. For this comparison, we have to multiply the estimate of M for MtDNA with a factor 4 to adjust for the four-fold lower population size. If we do that we find that *T. longispinosus* and *T. curvispinosus* have clearly higher migration rates for the nuclear markers (*T.l.* 32 vs. 4.6, *T.c.* 17.6 vs. 4.5), whereas for *P. americanus* the difference is not so large (6.5 vs. 3.4). Note that M for microsatellites depends on the average migration rates of males and females. Under the assumption of similar effective population sizes for males and females, the comparisons suggest that in the two host species males have migration rates that are between 6 and 15-fold higher than that of queens. In the monogynous parasite, this difference appears to be only three-fold.

### Methods to infer population structure

In our population genetic analysis we used several approaches: the newly introduced D statistic (Jost, 2008), the Bayesian clustering algorithm Structure (Pritchard *et al.*, 2000; Falush *et al.*, 2003) and the maximum likelihood estimates of mutation and migration rates based on coalescent theory (migrate-n by Beerli & Felsenstein (2001) and Migraine by Rousset & Leblois (2007).

The statistic *D* is by far the easiest of the methods we used, as it can simply be calculated from the data and it does not require elaborate computations. However, there are two main drawbacks of using *D*. First, it depends on both migration and mutation rates (Jost, 2008; Ryman & Leimar, 2009), which make observed differences between species or loci hard to interpret. Second, it only uses part of the data (namely the effective number of alleles, *n*_e_). The first drawback is not too severe for our dataset because we used the same loci for different species and found similar mutation rates between the loci we used. However, it makes comparisons between different marker systems (MtDNA vs. nuclear markers) difficult. We stress that whenever *D* values are compared between different loci, species or studies, it is necessary to consider the effect of mutation rates. The variability of the loci determines part of the outcome: very variable loci or long sequences lead to high *D* values, suggesting strong differentiation, whereas less variable loci or short sequences tend to underestimate structuring. Moreover, permutation tests are needed to determine whether *D* values are significantly higher than expected under panmixia.

We see no intuitive way to include genetic distances in the calculation of *D*, but this may not be necessary because there are elegant ways to include genetic distances in G_ST_-like statistics. We used γ_ST_ (see Fig. 5), which does not suffer from the problems that other heterozygosity-based G_ST_ statistics experience, because they use heterozygosity per nucleotide, which is naturally low.

We analysed the microsatellite data with Structure and found evidence for four clusters in each of the three species (see Fig. 6). Only in the host species *T. curvispinosus*, the Structure analysis reveals clear structuring. When comparing these Structure results with pairwise *D* values, we find good agreement. The subpopulations that are clustered together by Structure are mostly the ones that would also be clustered based on pairwise *D* values (averaged over loci). However, the neighbour-joining tree for *T. curvispinosus* shows that the clustering of subpopulations is not very strong: the tree is quite starlike and no two populations are really close to each other, but this information is not clear from the Structure analysis. For the data and the questions we have, the Structure analysis does not provide novel insights.

To estimate population wide mutation and migration rates, we used two programs, Migraine and migrate-n, which are both based on coalescent theory and which use a maximum likelihood framework to estimate population demographic parameters from genetic data from different locations. In principle, the two programs should find the same results. However, we found that for the microsatellite data Migraine performed better. For migrate-n, even when running the program for 1 000 000 steps and with adding heated chains, outcomes still depended on starting values for the MCMC chain and the program did not converge. We did not experience this problem for the MtDNA data. Results from Migraine and migrate-n will always be biased because of model misspecification. For example, we do not know how many subpopulations there really are in the species we study, and we chose to ignore all unsampled populations in the statistical model. Model misspecification is a difficult problem, but several studies suggest that inference can still be made (Beerli, 2004; Slatkin, 2005; Rousset & Leblois, 2007). Additionally, we can hope that the species we study are similar enough, so that we misspecified the model in the same way for each of the three species and results are therefore comparable.

Theoretical studies suggest that population size and migration rates are key parameters in coevolutionary arms races, because these parameters together determine how many new beneficial mutations enter a population in a given generation (Gandon & Michalakis, 2002). In this study, we compared genetic variation and population structure between a social parasite and its two main host species. We find similarities and differences and conclude that the three species probably do not differ much in terms of the evolutionary potential. However, we also find that the methods used in this and similar studies are necessarily based on simplified assumptions. For example, historical population size changes may have a larger impact on the observed genetic diversity than current effective population size does. If this is the case, then the observed genetic diversity may not be related at all to a species’ evolutionary potential. This is known for well-studied species such as *Drosophila melanogaster* and humans (Charlesworth, 2009, Karasov *et al.*, 2010) and it is likely to be the case for many other species. However, to take into account the demographic history of the species of interest, much more data are needed and more complex models would need to be used to estimate the relevant parameters.
